# Trigeminal Neuralgia due to Vertebrobasilar Dolichoectasia

**DOI:** 10.1155/2012/367304

**Published:** 2012-06-19

**Authors:** Wuilker Knoner Campos, André Accioly Guasti, Benjamin Franklin da Silva, José Antonio Guasti

**Affiliations:** ^1^Neuron, Institute of Neurosurgery, Baia Sul Medical Center, Florianopolis, SC, Brazil; ^2^Department of Neurosurgery, Bonsucesso Federal Hospital, Rio de Janeiro, RJ, Brazil

## Abstract

We presented a case of drug-resistant trigeminal neuralgia attributed to vertebrobasilar dolichoectasia, a rare condition characterized by enlargement, tortuosity, or elongation of intracranial arteries. Dolichoectatic vessels can cause dysfunction of cranial nerves through direct vascular compression. The relationships of vertebrobasilar dolichoectasia with the particularities of neurovascular conflict and images findings are discussed.

## 1. Introduction

Trigeminal neuralgia (TN) is a well-known clinical entity characterized by paroxysmal hemifacial pain [1]. Vertebrobasilar dolichoectasia (VBD) is a very unusual cause of TN associated to vascular compression due to characteristic conformation of VBD [2]. In the present study, we describe a patient who developed trigeminal neuralgia caused by VBD and was successfully treated by microvascular decompression (MVD).

## 2. Case Presentation

**Figure 1:**
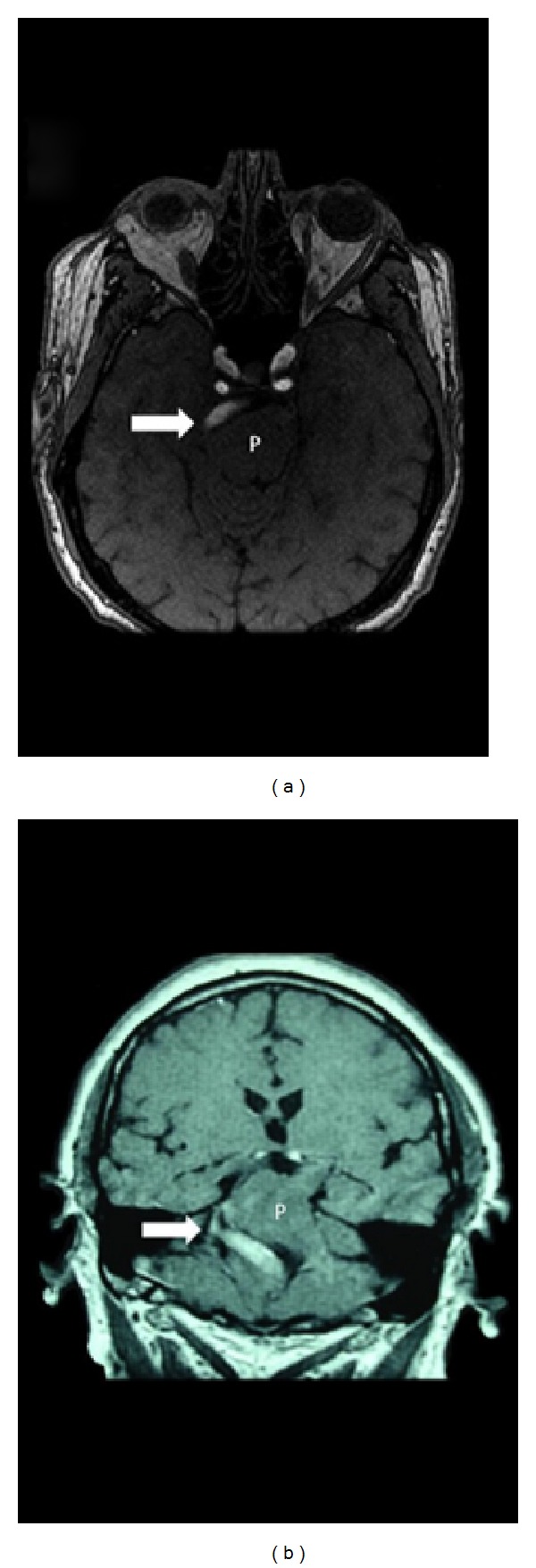
Neurovascular conflict: (a) (axial) and (b) (coronal) MRI reconstruction from a 3D-FSPGR study shows a dolichoectatic basilar artery (arrow) crossing and displacing upper pons (P) with compression of the right trigeminal nerve at the root entry zone.

**Figure 2:**
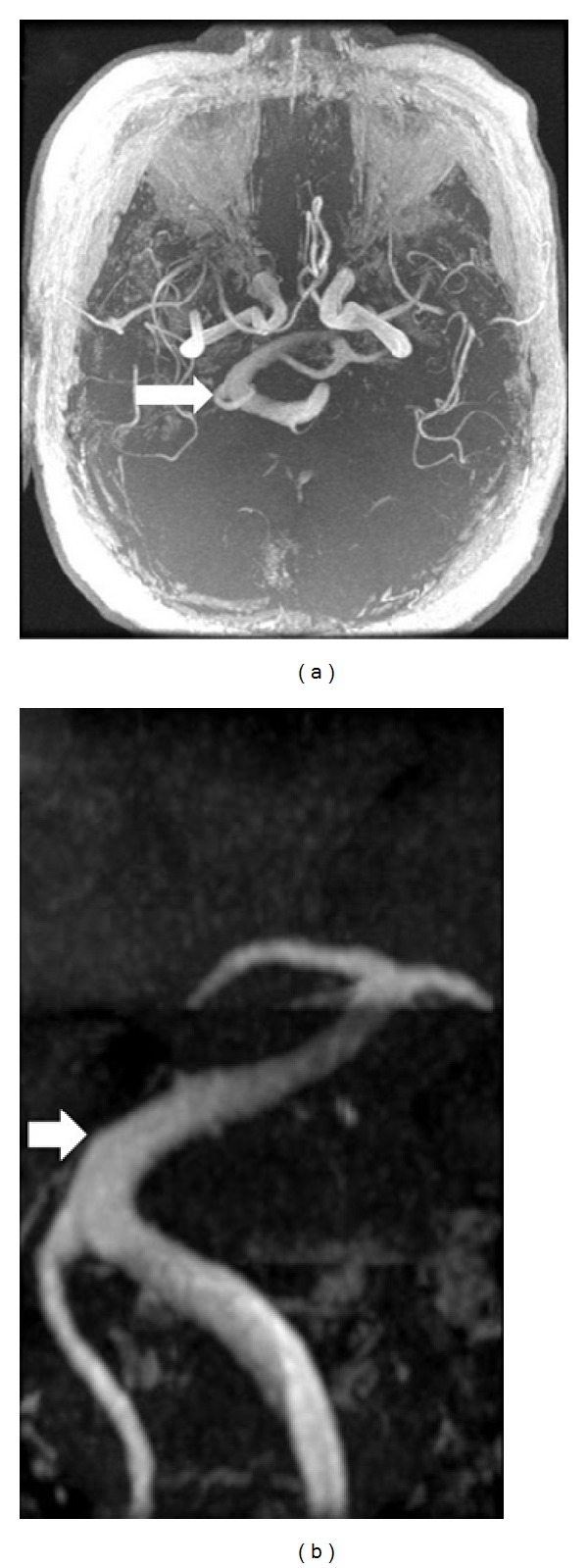
(a) (Axial) and (b) (coronal) magnetic resonance angiogram image shows enlarged and tortuous basilar artery. The basilar artery is moved more than 10 mm to the right (arrow).

A 63-year-old nondiabetic, nonsmoker, hypertensive male patient, who presented with a 3-year history of severe paroxysmal and lancinating right facial pain in V2 and V3 trigeminal territories. The pain used to come in sudden bursts lasting 1–5 minutes and recurs 10–20 times a day. The pain was not satisfactory controlled by oral opioids, tricyclic, or dual antidepressant. On physical examination, facial trigger points in the right maxilar region have been found without other neurological findings. Magnetic resonance imaging (MRI) and magnetic resonance angiogram (MRA) of the brain demonstrated an elongated and tortuous vertebrobasilar artery causing mechanical compression at the right trigeminal nerve root (Figures 1 and 2).

Surgical procedure (MVD) was then proposed due to refractoriness and images findings. A right retrosigmoid approach was performed with cerebellopontine angle exposure. Arachnoid dissection revealed a large vascular structure, identified as dolichoectasia of the basilar artery, dislocating and compressing the right ventrolateral region of brainstem and its respective trigeminal nerve root. As soon as the neurovascular conflicting area was identified, MVD technique placing pieces of Teflon between the trigeminal nerve and the basilar artery with displacement of dolichoectatic artery was performed. Care was taken not to injury of the artery during the vascular microdissection, because potentially fragile vessel walls in VBD.

A very satisfactory surgical decompression result was achieved and pain attacks ceased immediately and completely after surgery. During the follow-up period (24 months), the patient has reported excellent relief of pain and currently does not need any more medications.

## 3. Discussion

Intracranial arterial dolichoectasia is a condition characterized by enlargement, tortuosity, or elongation of major arteries at the base of the brain. The most common localization of dolichoectasia is the vertebrobasilar system [3]. Vertebrobasilar system is considered to be elongated if the basilar artery lies lateral to the margin of the clivus or dorsum sellae or if it bifurcates above the plane of the suprasellar cistern. Ectasia is considered to be present if the basilar artery has a diameter greater than 4.5 mm (Figures 1 and 2) [4]. 

The degeneration of the vascular wall due to atherosclerosis in association with hypertension is suggested as the pathogenic factor. However, other authors consider dolichoectasia to be a congenial vascular anomaly on the basis of histological observations of defect in the internal elastic lamina and thinning of the media secondary to smooth muscle atrophy [5]. In fact, dolichoectasia seems to be due to a congenital anomaly, and its evolution may be influenced by arterial hypertension and superimposed atherosclerosis. In the present case, we have agreed that the VBD origin was multifactorial. 

Two types of symptoms were found associated with intracranial arterial dolichoectasia: those resulting from the compression of structures adjacent to the abnormal vessel and those resulting of ischemic events. Trigeminal and facial nerves are the commonest cranial nerves involved [6]. However, direct compression by VBD is a uncommon cause of TN with an estimated general incidence of approximately 1% [4]. In patients with VBD, the compression has a slowly progression, so the brainstem can functionally tolerate severe distortion without overt clinical manifestations, which may explain why most patients with VBD are asymptomatic [7].

The proposed mechanism for TN is vascular compression at a specific portion of the cisternal segment of the nerve known as the root entry zone (REZ). There have been suggested that REZ is particularly vulnerable to continued pulsatile pressure, which may result in focal demyelination and “short-circuiting” of impulses [8]. Traditionally, the surgical options for patients with medically refractory pain include percutaneous or microsurgical rhizotomy and microvascular decompression (MVD). However, based on neurovascular conflicting, MVD has been practiced for the treatment of patients with/without TN associated to dolichoectatic artery [9, 10].

In fact, decompression of the nerve root produces rapid relief of symptoms in most patients with neurovascular conflicting, probably due to the resulting separation of demyelinated axons and their release from focal distortion reduce the spontaneous generation of impulses and prevent their ephaptic spread [11]. Interesting questions may be raised about this biological rationale like why immediate pain relief usually occurs after MVD if there is damage to neuronal structures? Likely the pathogenesis of TN is multifactorial and variably includes neurovascular contact, electrophysiological disruption of trigeminal circuits, or both.

Recent technological advancement in radiosurgery has revolutionalised all traditional surgical approaches in patients with TN. To date, Gamma knife surgery has become a keyhole to the minimally invasive approaches to TN associated or not with VBD. However, authors have shown that pain control rates of Gamma knife surgery in patients with TN associated with VBD were inferior to those of patients without VBD [12]. This evidence also comes in support of essential hole of neurovascular contact in the TN origin.

Two types of high-resolution MRI are interesting to study neurovascular contact in patients with TN: 3D-FIESTA (T2-weighted MRI) and 3D-FSPGR (contrast-enhance T1-weighted MRI). First sequence usually is better to demonstrate cranial nerves and their cisterns, while the others show better vascular structures [13, 14]. In fact, both high-resolution MRI sequences are complementary to demonstrate neurovascular contact in patients with TN. In the present case, although the patient had initially presented TN, the most important sequence was 3D-FSPGR because have they shown emphatically an aberrant vessel (VBD) and its relationship with brainstem. In additional, neurovascular conflict was evident due to enlargement of basilar artery and its compression on REZ (Figure 1). A subsequent angiogram was performed to study the details of VBD (Figure 2). Therefore, 3-D reconstructions from two types of high-resolution MRI are very useful for creating preoperative simulations and in deciding whether to perform surgery in patients with TN, mainly if associated to VBD [15].

The natural history of VBD shows that patients with VBD may experience mainly cerebrovascular event with high incidence after the initial diagnosis. This may be explained by the fact that there are various mechanisms by which VBD may promote brain ischemia, including occlusion of small perforating vessels, reduction of anterograde flow in the dilated artery, distortion and stretching of the branches of the basilar artery (Duret's Hemorrhages), and superimposed atheromatous changes [16]. This information is very essential in patient outcome with TN caused by VBD because of natural tendency to overvalue facial pain instead of dolichoectasia and its potential complication. In the present case, MVD was a safe and effective treatment for TN and the patient has been followed for other VBD symptoms or TN recurrence.
